# Nirvana

**Published:** 1889-06

**Authors:** 


### NIRVANA.

The Ancients taught that the whole universe was permeated and sustained by the Creative Power, that each world and atom received its impulse from that divine source, and that everything in existence was subject to the purifying process of the Divine Element. They also believed that each world and atom contained the Aetheric essences of the four elements Earth, Air, Fire and Water, in a state of more or less activity, and that as the vibratory presence of these different elements was more or less defined, was each atom compelled to conform to the laws of atomic association pertaining to the mineral, vegetable, and animal and human kingdoms. The mineral, vegetable and, animal were subject to and controlled by those impulses pertaining to the forces ruling that relatively distinct portion of the universe. Each atom had a vibratory condition which defined its growth and operative power while evolving through each of the several departments, and could not reach beyond its present sphere of action until it became conscious or equal to all manifestations connected with the realm to which it had so far attained. This evolving, purifying process attached new vibratory conditions adapting the atom to a new realm, and so on until it became fully active.

They believed that the bodies of animals consisted of atoms that had become sublimated by contact with the lower orders of existence, and had therefore attained elements actively operating in harmony with the animal. Human life, they considered to exist in harmony with a relatively more extensive combination of forces identified with the great Universal Creative Will. The atoms of a human body, therefore, must have become more refined and intensely active to become posited in contact with the subtle elements that sustain human life on this planet. The Ancients recognized in man the possibility of growing into knowledge of the subtle Aetheric forces in which he exists and also the ability to control and operate to a great extent such of those forces as relate to the strata of life below him. They also sensed that the human mind, when active in the recognition of the wonders of Creative Power, would become full of faith, and in humility live in accord with Divine wish. All Saviours and Kingly minds must have attained that condition, or they could not have demonstrated kingly powers, which require true conception of the Divine of self. The more we become knowing to the wonders of God and He becomes present and operative with us, the more of his Creative Will becomes a part of us ; and relatively we sink more and more into the bosom of Infinity or God conciousness. A perfect attainment to God conciousness would find us lost in God and, in a certain sense, self-annihilation would take place. The Hindu state of Nirvana, which many have construed imperfectly to represent a literal self-annihilation, on the contrary, is a state of Supreme conciousness and attunement with Divine Will. The annihilation inferred consists in the destruction and subjugation of lower self; the complete mastery of every element ruling lower orders of existence. The process of Spiritual growth demands the practice of all virtues and subjugation of all vices. All Human progress is the gradual growth toward the Hindu state of Nirvana. Until man rests in the state of Nirvana or becomes fully conscious in his Spiritual nature, he cannot exist in the true presence of God and truly make the Father one with him.—GALTO, in "Greeley."

